# Full-length transcriptional analysis reveals the complex relationship of leaves and roots in responses to cold-drought combined stress in common vetch

**DOI:** 10.3389/fpls.2022.976094

**Published:** 2022-09-23

**Authors:** Xueyang Min, Qiuxia Wang, Zhenwu Wei, Zhipeng Liu, Wenxian Liu

**Affiliations:** ^1^State Key Laboratory of Grassland Agro-Ecosystems, Lanzhou University, Engineering Research Centre of Grassland Industry, Ministry of Education, Western China Technology Innovation Centre for Grassland Industry, College of Pastoral Agriculture Science and Technology, Lanzhou University, Lanzhou, Gansu, China; ^2^College of Animal Science and Technology, Yangzhou University, Yangzhou, Jiangsu, China

**Keywords:** *Vicia sativa* L, combined abiotic stress, leaves, roots, full-length transcripts

## Abstract

Plant responses to single or combined abiotic stresses between aboveground and underground parts are complex and require crosstalk signaling pathways. In this study, we explored the transcriptome data of common vetch (*Vicia sativa* L.) subjected to cold and drought stress between leaves and roots *via* meta-analysis to identify the hub abiotic stress-responsive genes. A total of 4,836 and 3,103 differentially expressed genes (DEGs) were identified in the leaves and roots, respectively. Transcriptome analysis results showed that the set of stress-responsive DEGs to concurrent stress is distinct from single stress, indicating a specialized and unique response to combined stresses in common vetch. Gene Ontology (GO) enrichment analyses identified that “Photosystem II,” “Defence response,” and “Sucrose synthase/metabolic activity” were the most significantly enriched categories in leaves, roots, and both tissues, respectively. The Kyoto Encyclopedia of Genes and Genomes (KEGG) enrichment analysis results indicated that “ABC transporters” are the most enriched pathway and that all of the genes were upregulated in roots. Furthermore, 29 co-induced DEGs were identified as hub genes based on the consensus expression profile module of single and co-occurrence stress analysis. In transgenic yeast, the overexpression of three cross-stress tolerance candidate genes increased yeast tolerance to cold-drought combined stress. The elucidation of the combined stress-responsive network in common vetch to better parse the complex regulation of abiotic responses in plants facilitates more adequate legume forage breeding for combined stress tolerance.

## Introduction

Abiotic stress conditions, including drought, heat, cold, salinity, oxidative stress, and nutrient deficiency, are the primary cause of yield loss and quality reduction worldwide (Hasanuzzaman et al., 2019; Gong et al., 2020). Previous research has shown that the combination of multiple stresses could cause a more significant negative impact on crop productivity than the stress applied individually (Gargallo-Garriga et al., 2015; Correia et al., 2018). Under natural and cultivated field conditions, drought is usually combined with abiotic and biotic stresses. Although these stressors were randomly combined, the combinations of drought with cold stress occur frequently in cold, arid, and semiarid regions. When the frozen up ground tissues are subjected to the sun, their water potential will become higher than that of the surrounding air, and the frozen soil will increase water flow, which results in the leaves’ water loss and restricts the water uptake and transport from the underground to the xylem (Suzuki et al., 2014; Zandalinas et al., 2018). Thus, to improve crop yield and quality, it is crucial to understand the mechanisms underlying plant responses to combined stress challenges.

Previous reports have provided a plethora of information on plants exposed to single abiotic stress, while these studies cannot be used to obtain the effects of a combination of stresses on plants (Suzuki et al., 2014; Correia et al., 2018; Mota et al., 2021). Currently, an increasing number of reports have revealed the combination of abiotic stress conditions at physiological and molecular levels (Mittler, 2006; Choudhury et al., 2017). These studies showed that the combined stress could impose a specific set of requirements, and required the tailoring of unique and shared regulatory transcript pathways including photosynthesis, hormone signaling, antioxidant mechanisms, and transcription factors (Mahalingam, 2015; Zhu, 2016). Under natural conditions in northeast and northwest China, maize often suffers from combined low temperature and drought stress at the seedling stage. The authors used transcriptomic and metabolomic analyses to systematically analyze the differences in the phenotype and photosynthetic physiology of maize seedlings under single drought, chilling and combined stress conditions and found that drought stress could significantly alleviate the damage caused by chilling stress under combined stress (Guo et al., 2021). In addition to transcriptomic-based technology, a novel system for evaluating drought-cold tolerance by using a chlorophyll fluorescence model was established in grapevine, and find that the temperature of 50% electrolyte leakage values exhibited good reliability for evaluating the grapevine tolerance to drought-cold stress (Su et al., 2015). Furthermore, the source (leaves) and sink (roots) tissues are two distinct resource pools and are linked through the vascular system. When plants suffer adverse conditions, they rapidly perceive environmental signals and then function in different organs to survive (Werner et al., 2008; Lozano-Elena et al., 2022). Various signaling molecules have been shown to play vital roles in response to abiotic stress in leaves and roots, such as phytohormones, calcium, carbohydrates, reactive oxygen species (ROS), and photosynthetic electrons (Zhang and Sonnewald, 2017; Min et al., 2020b,c). Nevertheless, systematic comparisons among leaves and roots under combined drought and cold stress are still limited. Thus, recognizing the integration of changes under combined stress, and possible intercommunication and specific mechanisms between aboveground and underground tissues, are essential to expanding our knowledge of plant responses to different stress stimuli coordinately in different tissue types.

Common vetch is an economically important annual legume species that is commonly cultivated in semiarid regions as a cool-season forage legume due to its rich nutritional value, low requirement, suitable intercrops for reducing diseases, ability to improve soil properties, and superior abiotic stress tolerance (Larbi et al., 2011; Huang et al., 2017; Kartal et al., 2020). Compared to some model plants, common vetch often has a more complex genetic basis and is likely to have additional mechanisms to adapt to stress stimuli. In this study, we investigated the transcriptional dynamics elicited by drought in combination with cold between leaves and roots *via* a meta-analysis of our previously produced common vetch reference transcriptome database to discover combined stress-responsive machines and core regulatory genes (Min et al., 2020c). We also evaluated the function of the cross-stress genes in transgenic yeast. The present study will facilitate the identification of unique and common regulators between leaf and root tissues and consequently support the improvement of abiotic stress tolerance in legumes.

## Materials and methods

### Sample collection and data acquisition

Experimental materials, seed pretreatment, and seed germination were performed as previously described (Min et al., 2020b). The control groups were hydroponically cultured with 1/2 MS (half-strength Murashige and Skoog, pH = 5.8) solution under plant incubator conditions (20°C, 16 h/8 h (light/dark) photoperiod, 80 μmol m^−2^ s^−1^ photosynthetically active radiation and 70% relative humidity) for 7 days. To conduct cold-drought combined stress, seven-day-old uniformly growing seedlings were transplanted into 1/2 MS solution containing 20% PEG solution to simulate drought stress while adjusting the temperature of the plant growth incubator to 4°C, the 1/2 MS treated plants at 20°C were used as control. Our previous studies found that short-term cold-drought stress could cause the aboveground of common vetch to wilt, and the malondialdehyde and soluble sugar contents were significantly increased at 24 h under 4°C in leaves (Min et al., 2020b,c). Furthermore, to avoid the influence of different photoperiods on experimental materials, samples from the control and cold-drought combined conditions were grown in parallel and harvested after 24 h of treatments. After 24 h, the third and fourth leaves from the top of the plants and root tips (~1.5 cm) of four seedlings were harvested and pooled together. Twelve samples [(control and 4°C + 20% PEG for 24 h) × two tissues (leaves and roots) × three replications] were used for sequencing analysis, including 0 and 24 h two-time points. RNA isolation, library construction, and sequencing were performed as previously described (Min et al., 2020b). Finally, bowtie2 is used to map the clean reads from a common vetch Illumina data set to a reference transcript.^1^

### Full-length reference transcriptome construct

Firstly, equal amounts of total RNA from leaves, stems, and roots under normal conditions, as well as leaves and roots treated with salt, low temperature, drought, and cold-drought combined stresses for 24 h with three biological replications were combined to construct a reference transcriptome by PacBio sequencing. Then, the raw reads were processed into error-corrected reads of insert (ROIs) using the ToFu pipeline with parameters of prediction accuracy > 0.75, full pass ≥ 0, and sequence length ≥ 300 bp. After that, the full-length, non-chimeric transcripts were determined by searching for the PolyA, and the 5′ and 3′ cDNA primers in ROIs. The Iterative Clustering for Error Correction (ICE) was used to cluster the consensus isoforms and full-length consensus sequences, and each cluster was polished with Quiver to obtain a consensus sequence, and full-length transcripts with post-correction accuracy above 99% were generated. Finally, the data of Illumina RNA-Seq were used to correct the low-quality isoforms, and the CD-HIT program was used to delete redundancy, with identity > 0.99.

### Expression profile analysis

Gene expression levels of each sample were calculated by RSEM (Li and Dewey, 2011), and the read count for each transcript was obtained from the mapping results. To obtain the difference in gene expression, the parameters of false discovery rate (FDR) < 0.01 and Log_2_ (fold change) > 2 were used to identify differentially expressed genes (DEGs) (0). The expression profile of DEGs was clustered by Short Time-series Expression Miner (STEM)^2^ software, with *p* ≤ 0.05, the maximum number of model profiles was 15, and the log normalizes the data. The specific primers were designed by Primer-BLAST progress on the NCBI website as shown in Supplementary Table S1 and synthesized by Sangon Biotech (Shanghai China). qRT–PCR was conducted as previously described (Min et al., 2020b). The *Vsactin* (*Unigene 68,614*) gene was used to calculate the relative expression levels following the 2^−△△CT^ method.

### Functional annotation and network analysis

To obtain the annotation information of the transcript, BLAST (version 2.2.26, *E* ≤ 10^−5^) was used to search against the NR, SwissProt, GO, COG, KOG, Pfam, and KEGG databases (Altschul et al., 1997). The GOseq R package and KOBAS software were used to conduct GO and KEGG enrichment (*p* < 0.05; Xie et al., 2008). PlantTFDB (v5.0) was used to identify transcription factors of common vetch (Jin et al., 2016). The “best hit in *Arabidopsis thaliana*” was checked to obtain the best hits. *Arabidopsis* as a reference to predict protein–protein interactions, protein function, and subcellular localization in STRING (V11.5)^3^. Then, the key nodes of the interaction network were modulated in Cytoscape.^4^

### Generating overexpression lines in yeast

The coding regions of *F01.PB9597* (*VsUMAMIT*), *F01.PB3916* (*VsPM19*), and *F01.PB22519* (*VsP5CS2*:) were amplified by primers as shown in Supplementary Table S1. The amplification product is purified by SanPrep Column DNA Gel Extraction Kit (Sangon Biotech, Shanghai). The purified cDNA products were directly cloned into the *pYES2* vector (Invitrogen, Carlsbad, United States) and then transformed into *E. coli* DH5α. The recombinant plasmid was sent to Sangon Biotech for sequencing by M13 universal primers. The following steps were performed according to the protocol described by Kawai et al. (2010). Freeing and osmotic (drought and salt) tolerance were performed in the SC-Ura medium, according to previously described methods (Min et al., 2020a). For simultaneous (cold-drought) stress treatment, yeast cells were grown in an SC-Ura liquid medium containing 30%, at −20°C for 36 h. Finally, the serial dilutions (1, 10^−1^, 10^−2^, 10^−3^, 10^−4^, and 10^−5^) were spotted onto SC-ura, agar plates, and carried out in triplicate at 30°C for 48 h.

### Statistical analysis

Pearson correlation and principal component analysis (PCA) of the expression level among each sample were calculated *via* the R cor.test function.^5^ Statistical significance was analyzed through Duncan’s multiple range test at a 0.05 level. Venn diagrams, heatmaps, and clusters were generated using TBtools (Chen et al., 2020).

## Results

### Illumina-Seq, assembly, and annotation

**Figure 1:**
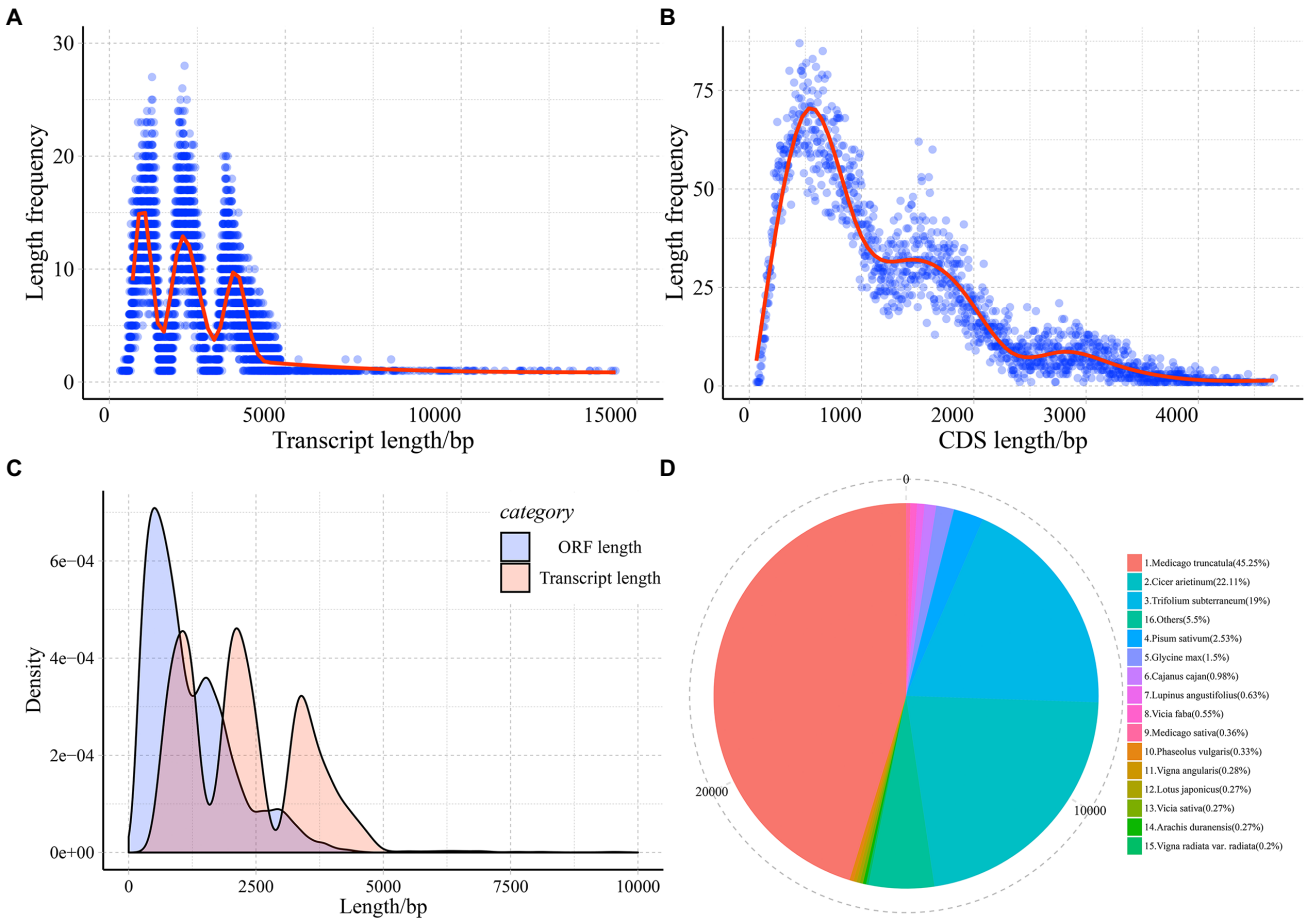
Length distribution of the assembled transcript **(A)**, CDS **(B)**, frequency distribution comparison of transcript and CDS **(C)**, and annotation distribution **(D)**.

The 12 cDNA libraries of common vetch treated by cold-drought combined stress conditionals between leaves and roots for 24 h (CDL1, CDL2, CDL3, CDR1, CDR2, CDR3) and normal conditionals (CKL1, CKL2, CKL3, CKR1, CKR2, CKR3). In total, 311.62 million high-quality reads were identified in common vetch. The percentages of average GC content and Q30 were 43.13 and 91.75, respectively. Bowtie2 software was used to map the reads onto the previously produced common vetch full-length transcript database. Overall, the average mapped read, uniquely mapped read, and multi-mapped read percentages were 76.82%, 26.99%, and 73.01%, respectively (Supplementary Table S2). Finally, we predicted a total of 30,727 non-redundant transcripts from a min length of 304 to 14,390 bp, the mean length was 2287.35 bp with an N50 of 3.73 kb (Figure 1A). The open reading frame (ORF) length ranged from 63 to 4,674 bp, with a mean length of 1,188.58 bp (Figures 1B,C). In total, 30,250 (98.45%) transcripts were functionally annotated, and 4,879 (15.88%) were annotated in all eight databases. A total of 521 (1.7%) genes were not annotated (Supplementary Table S3). Next, the BLASTP program was used to search against the NR database, examining the evolutionary relationships among common vetch and other plants. The results indicate a close relationship among Leguminosae plants, only 4% of the transcripts were annotated to plants outside the genus *Medicago* (Figure 1D).

### Identification and functional profiles of DEGs

**Figure 2:**
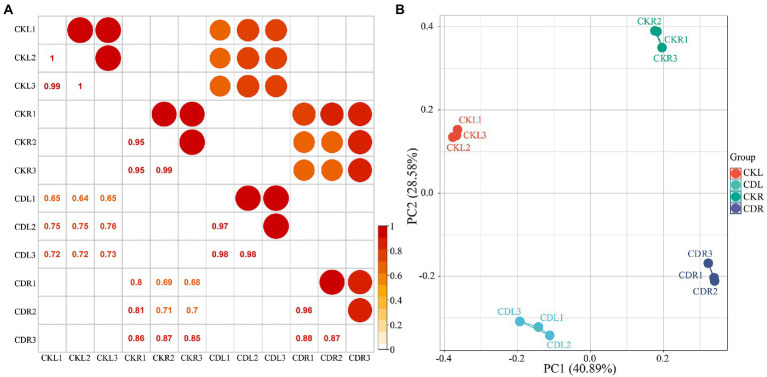
Pearson correlation **(A)** and principal component analysis **(B)** among 12 samples. CKL, leaf control groups; CKR, root control groups; CDL, PEG + cold treated leaf groups; CDR, PEG + cold treated root groups. The red background represents a high correlation coefficient.

The mapping results were combined with the RSEM method for accurate transcript quantification from RNA-Seq data (Li and Dewey, 2011). The FPKM (fragments per kilobase of transcript per million mapped reads) value was used to indicate the expression abundance of the corresponding transcript. When comparing three biological replicates, Pearson’s correlation indicated significant correlations (*p* < 0.05, *R^2^* ≥ 0.88). A significant positive relationship was found between normal and cold-drought combined treatments in the same tissues (*p* < 0.05, *R^2^* ≥ 0.64) but not in the different tissues (*p* > 0.05, *R^2^* < 0.05). Compared to the two tissues in response to abiotic stress, a more significant positive relationship was shown in roots (*p* > 0.05, *R^2^* with an average of 0.77), indicating that leaves were more sensitive to abiotic stress (Figure 2A). To understand how common vetch transcripts respond to cold-drought stress between leaves and roots, the mRNA populations were compared with PCA (Figure 2B), and the results showed that the four groups were separated from each other and the CK.

**Figure 3:**
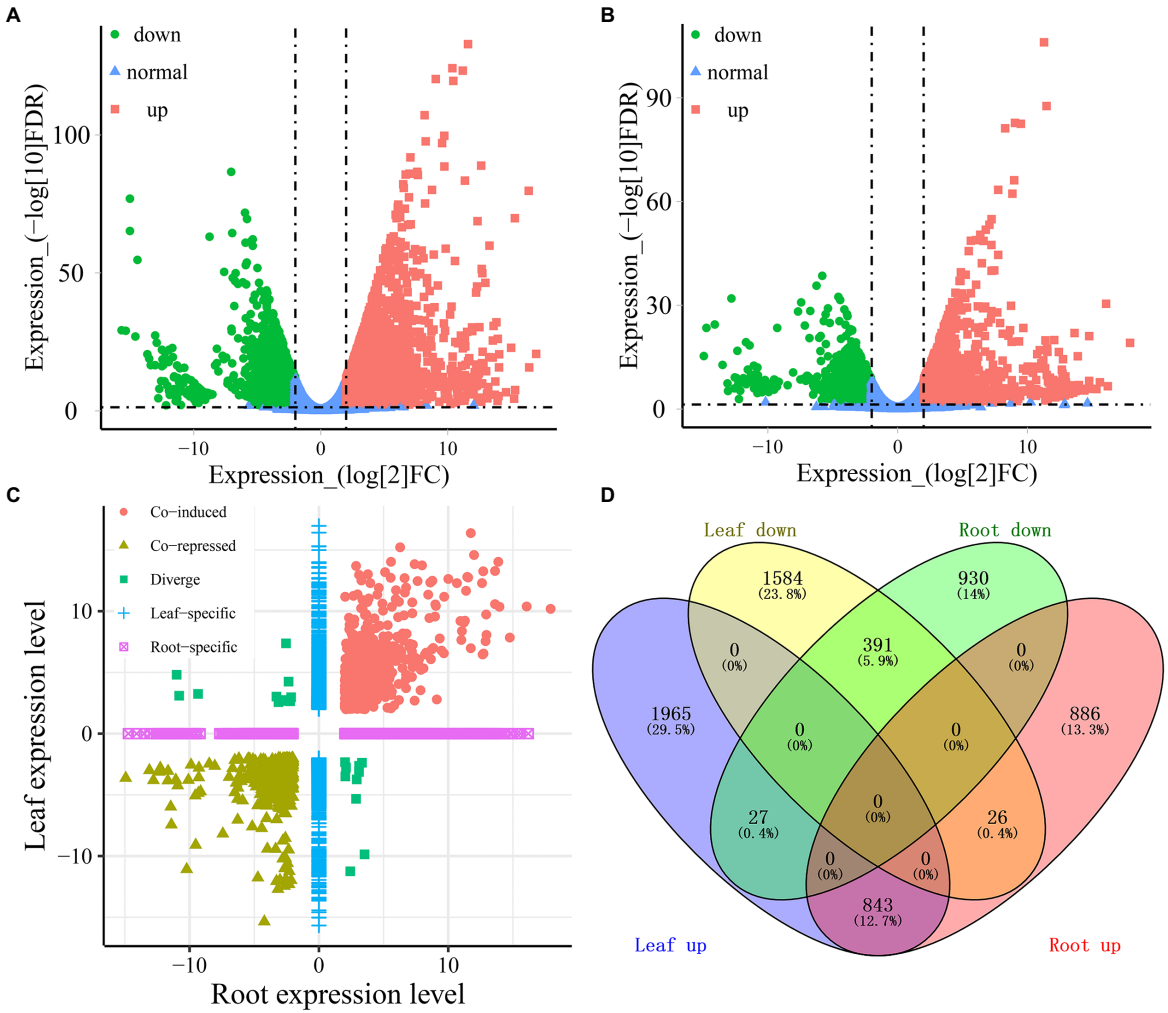
Identification of the DEGs in response to cold-drought stress between leaves and roots. Volcano plots display log_2_ converted fold changes and FDR values in leaves **(A)** and roots **(B)**. The expression level distribution **(C)** and the number of up-and downregulated DEGs **(D)** in each tissue.

A total of 4,836 and 3,103 DEGs were identified in the leaves and roots, respectively, under cold-drought combined conditions compared with the control. Among these DEGs, 2,835 and 1,755 genes were upregulated in leaves and roots, respectively, while 2,001 and 1,348 DEGs were downregulated in leaves and roots under cold-drought combined stress, respectively (Figures 3A,B). Among them, 3,549 (53.3%) and 1,816 (27.3%) DEGs were found to be leaf and root-specific, respectively (Figures 3C,D). Notably, a total of 1,187 (19.4%) genes were differentially expressed in both tissues; of them, 843 (12.7%) were co-induced, 391 (5.9%) were co-repressed and 53 (0.8%) were oppositely expressed in the leaves and roots (Figures 3C,D). For the 521 novel transcripts, their expression patterns were more likely to be downregulated under cold-drought stress (Supplementary Table S4). Among them, 41 and 18 downregulated DEGs were identified in leaves and roots, respectively. Twenty-five and 3 DEGs were classified as upregulated in leaves and roots, respectively. Furthermore, 12 co-induced and 8 co-repressed DEGs were identified in both leaves and roots (Supplementary Figure S1).

To further confirm the reliability of our RNA-Seq data, 15 candidate DEGs were selected for qRT-PCR validation (Supplementary Figure S2). As shown in Supplementary Figure S3, the expression levels of these DEGs in both leaves and roots were significantly correlated with the FPKM values (R^2^ = 0.8). Among them, one (*F01.PB19254*) was downregulated, one (*F01.PB13497*) was oppositely expressed, and the remaining genes were upregulated after cold-drought treatment.

### Functional enrichment analysis of potential DEGs

#### Consensus and different co-expression modules identify by GO enrichment analysis

**Figure 4:**
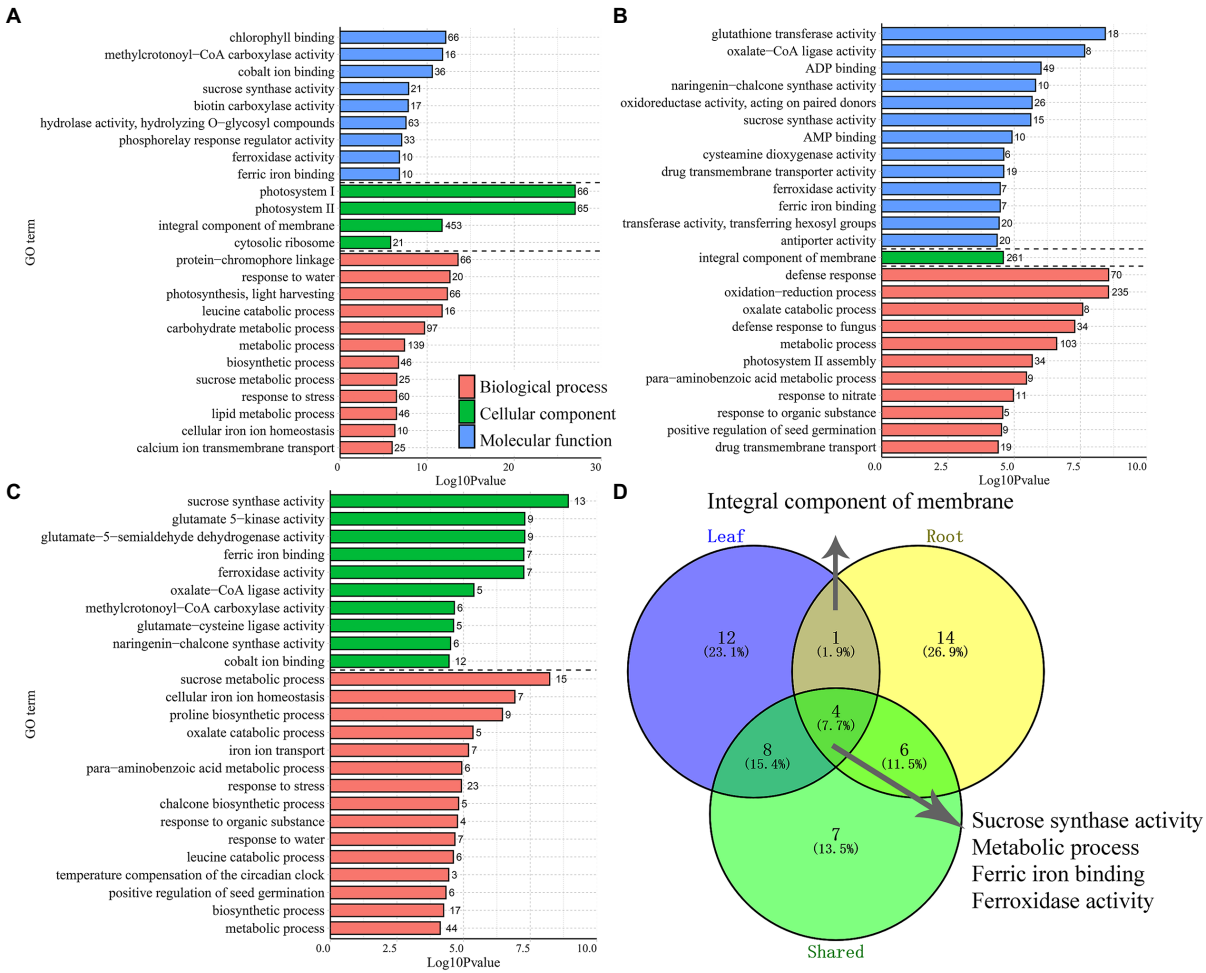
The distribution of the top 25 most represented GO categories. The DEGs obtained from leaves **(A)**, roots **(B)**, and shared between two tissues **(C)** were assigned to three main categories: biological process, cellular component, and molecular function. The number of enriched GO categories distribution **(D)**.

In plants, leaves and roots can respond asymmetrically under abiotic stress, as has been observed at the physiological, morphological, and genetic levels. However, the coordinated variations in the functional genes of different tissues were also observed in common vetch. GO enrichment results showed that 12 and 14 GO enriched terms were specifically divided into leaves and roots, and five GO terms shared by leaves and roots, including “Sucrose synthase activity,” “Metabolic process,” “Ferric iron binding,” “Ferroxidase activity,” and “Integral component of membrane” (Figure 4D).

Plant leaves and roots are tightly linked and jointly respond to fluctuating environments. Even though above-and below-ground follow distinct developmental trajectories, some highly coordinated biological processes also exist. GO enrichment (top 25, *p* < 0.05) found that cold-drought combined treatments significantly affected 12 BPs (biological process), 9 MFs (molecular function), and 4 CCs (cellular component) in leaves (Figure 4A). The “Photosystem II” was the most significantly enriched category in leaves, followed by “Protein-chromophore linkage” and “Response to water.” Among the 67 “Photosystem II” term-enriched DEGs, all of them were upregulated in leaves and showed tissue-specific expression patterns, and 61 of them were not detected in roots (Supplementary Table S5). Notably, 20 DEGs enriched in the “Response to water” term were all upregulated, and 40% of DEGs were significantly upregulated (Log_2_FC > 10) in leaves. One, four, nine, and six DEGs of them were annotated as “Endochitinase A2,” “Embryogenic cell protein 40,” “Dehydrin DHN2,” and “Dehydrin-cognate,” respectively. Nearly half of them were also upregulated in roots (Supplementary Table S5).

Similarly, 11 BP, 13 MF, and one CC category were enriched in the roots. The three most significantly enriched categories were “defence response,” followed by “oxidation–reduction process” and “glutathione transferase activity” (Figure 4B). In total, 70 DEGs were enriched in the “defence response” term, 53 (88.6%) were downregulated, and 46 specifically expressed DEGs were found in roots, with 8 upregulated and 38 downregulated. In particular, the DEGs annotated as “TMV resistance protein” and “NB-ARC domain disease resistance protein” were mostly downregulated, implying that DEGs related to “defence response” might play a negative role in the response to multiple stresses. The DEGs belonging to “ABA-responsive protein” and “Endochitinase A2” were all upregulated. Notably, 18 DEGs enriched in the “glutathione transferase activity” term was all upregulated in roots, among which five were upregulated in the leaves and roots (Supplementary Table S6). Furthermore, there were 1,187 DEGs were enriched into 15 BP and 10 CC categories in both tissues (Figure 4C). The “Sucrose synthase/metabolic activity” terms were most significantly enriched, followed by “Glutamate-5-semialdehyde dehydrogenase activity,” and “Glutamate 5-kinase activity.” Interestingly, 24 DEGs were detected in the above-mentioned terms, and all of them were upregulated in the leaves and roots, indicating that these DEGs may be highly coordinated in response to abiotic stress between the above-and underground tissues of common vetch (Supplementary Table S7).

#### Consensus and different co-expression modules identify by pathway enrichment

**Figure 5:**
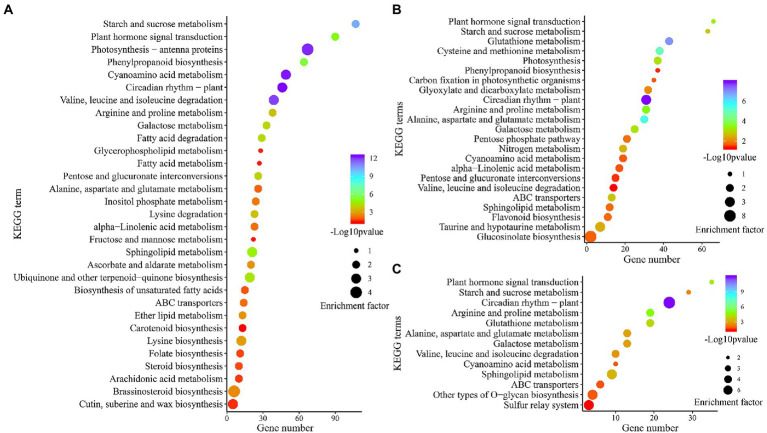
Scatter diagram of significantly (*p* < 0.05) enriched KEGG pathways of DEGs in leaves **(A)**, roots **(B)**, and DEGs differentially induced in both tissues **(C)**.

To explore the key pathway under combined stress, KEGG pathway analysis of DEGs obtained from leaves, roots, and co-induced (shared in both tissues) was conducted by meta-analysis. The enrichment result showed that 1,161, 881, and 340 cold-drought-induced DEGs were assigned to 121, 112, and 88 pathways in the leaves, roots, and co-induced, respectively. The significantly enriched pathways (*p* < 0.05) were further screened, and it was found that 10 common elements (23.3%), including “Circadian rhythm-plant,” “Cyanoamino acid metabolism,” “Valine, leucine, and isoleucine degradation,” “Starch and sucrose metabolism,” “Plant hormone signal transduction,” “Sphingolipid metabolism,” “Galactose metabolism,” “Arginine and proline metabolism,” “ABC transporters,” and “Alanine, aspartate and glutamate metabolism,” were the shared pathways in leaves (Figure 5A), roots (Figure 5B), and co-induced DEGs (Figure 5C). Except for these coenriched pathways, 18 (41.9%), 9 (20.9%), and 2 (4.7%) pathways were specifically enriched in leaves, roots, and co-induced DEGs, respectively.

KEGG pathway enrichment results showed that “ABC transporters” was the most enriched pathway among leaves, roots, and DEGs shared between both tissues. In the current study, six common vetch ABC transporters (*VsABC*) were detected in both tissues, and all of them were upregulated in roots, while, three of them had the opposite expression patterns (*F01.PB27856*, *F01.PB32848*, and *F01.PB36915*), which were upregulated in roots, but downregulated in leaves (Supplementary Table S8). Eight and seven *VsABC* genes showed tissue-specific expression profiles, which were specifically identified in leaves and roots, respectively. Compared with roots, more ABC transporters were downregulated in leaves, suggesting that the function of *VsABC* genes may have tissue specificity, and play different functions between underground and aboveground organizations.

Previous studies have found that the *OsABCG9* gene is primarily expressed in rice leaves during vegetative growth. In mutated *OsABCG9* plants, the cuticular wax contents on the leaves are diminished by half, and this mutant exhibited growth retardation and sensitivity to drought stress (Nguyen et al., 2018). It is worth noting that the pathway of “Cutin, suberin, and wax biosynthesis” was significantly enriched in leaves. Five DEGs were identified in this pathway, two of which were upregulated in both tissues (*F01.PB14194*: *Protein ECERIFERUM 1* and *F01.PB19883*: *Cytochrome P450 94A1*), one upregulated (*F01.PB22120*: *Cytochrome P450 86A1*) in leaves, and reaming two (*F01.PB25896* and F01.PB31997 were identified as *Fatty acyl-CoA reductase 1*) downregulated in leaves (Supplementary Table S8), indicating that *VsABC* genes may play an important function by regulating the common vetch cutin, suberin, and wax biosynthesis pathway against combined abiotic stress in leaves.

### Transcription factors in response to cold-drought stress

**Figure 6:**
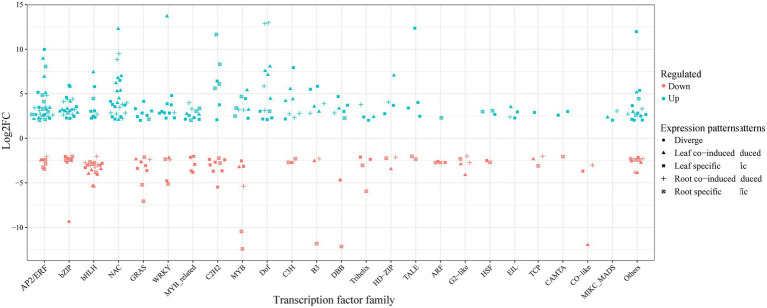
The expression pattern distribution of the most enriched transcription factors in response to cold-drought stress between leaves and roots.

Transcription factors are important upstream regulators, directly responsible for the activation or repression of various abiotic stresses (Adcock and Caramori, 2009). For example, ERF (ethylene-responsive element binding proteins), bZIP, NAC (NAM/ATAF/CUC), GRAS (GAI/RGA/SCR), bHLH (basic helix–loop–helix), WRKY (WRKY-domain), and MYB (myeloblastosis) have been proven to participate in multiple stresses in various plants (Gujjar et al., 2014; Baillo et al., 2019). Consistent with previous studies, 189 and 130 DEGs were identified as transcription factors under cold-drought treatments in common vetch leaves and roots and were further divided into 37 and 32 families, respectively (Supplementary Table S9). ERF, bZIP, bHLH, NAC, GRAS, and WRKY represented the most abundant families. Compared with other families, the members of NAC, Dof, EIL, AP2, and MIKC-MADS were all upregulated in both tissues. Furthermore, 126 (49.2%) and 62 (24.2%) tissue-specific induced TFs were identified in leaves and roots, respectively (Figure 6). Some tissue-specifically expressed TFs have also been identified and have less-defined roles in abiotic stress responses. For example, GATA, GRF, M-type_MADS, NF-YA, NF-YC, Nin-like, and YABBY were specifically identified in leaves, FAR1 and SRS were specifically identified in roots, perhaps because functional differentiation occurred in the long-term evolutionary process, and different complementary responses of above-and belowground tissues to combined abiotic conditions.

We also identified sets of TFs with similar behaviors among leaves and roots during cold-drought combined stress, and the most abundant TF families were the NAC (9), followed by the ERF (8), bZIP (7), bHLH (6), and MYB (5) families. Among them, 63 co-responsive DEGs were identified in both tissues, which were classified into 24 TF families (Supplementary Figure S4), including 45 and 17 DEGs that were up-and downregulated, respectively. Only one gene (F01.PB17105: bZIP) showed the opposite expression pattern, which was upregulated in leaves but downregulated in roots.

### Consensus module identification and hub gene protein–protein interaction network analysis

**Figure 7:**
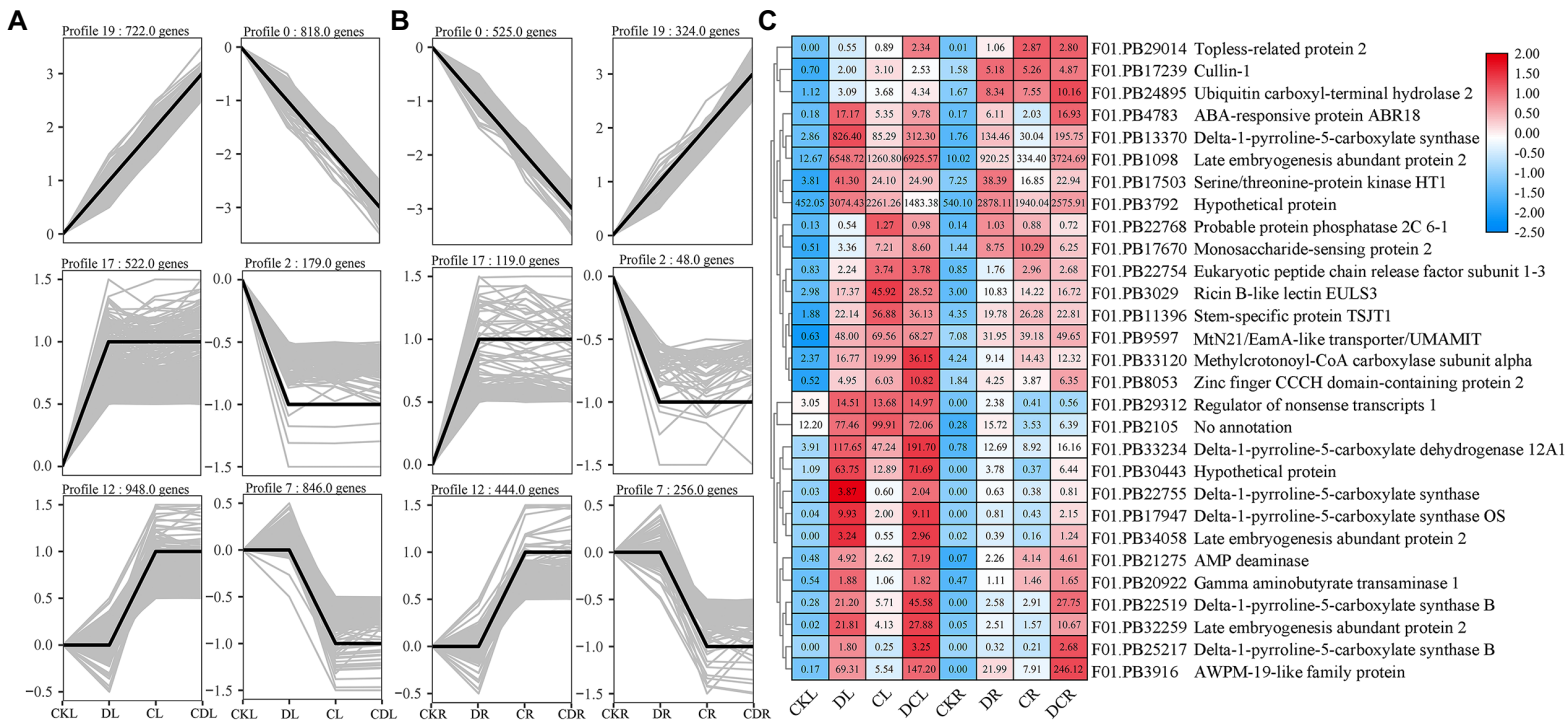
Dynamic progression of the common vetch transcriptome under cold-drought stress. **(A,B)** show the dynamic expression of DEGs in leaves and roots, respectively, by K-means clustering. **(C)** shows the expression profile of shared DEGs in profile 17 between leaves and roots. CKL and CKR are leaf and root control groups, respectively, DL, CL, and DCL are leaf under single drought, single cold, and cold-drought combined stress respectively, DR, CR, and DCR are root under single drought, single cold, and cold-drought combined stress, respectively.

**Figure 8:**
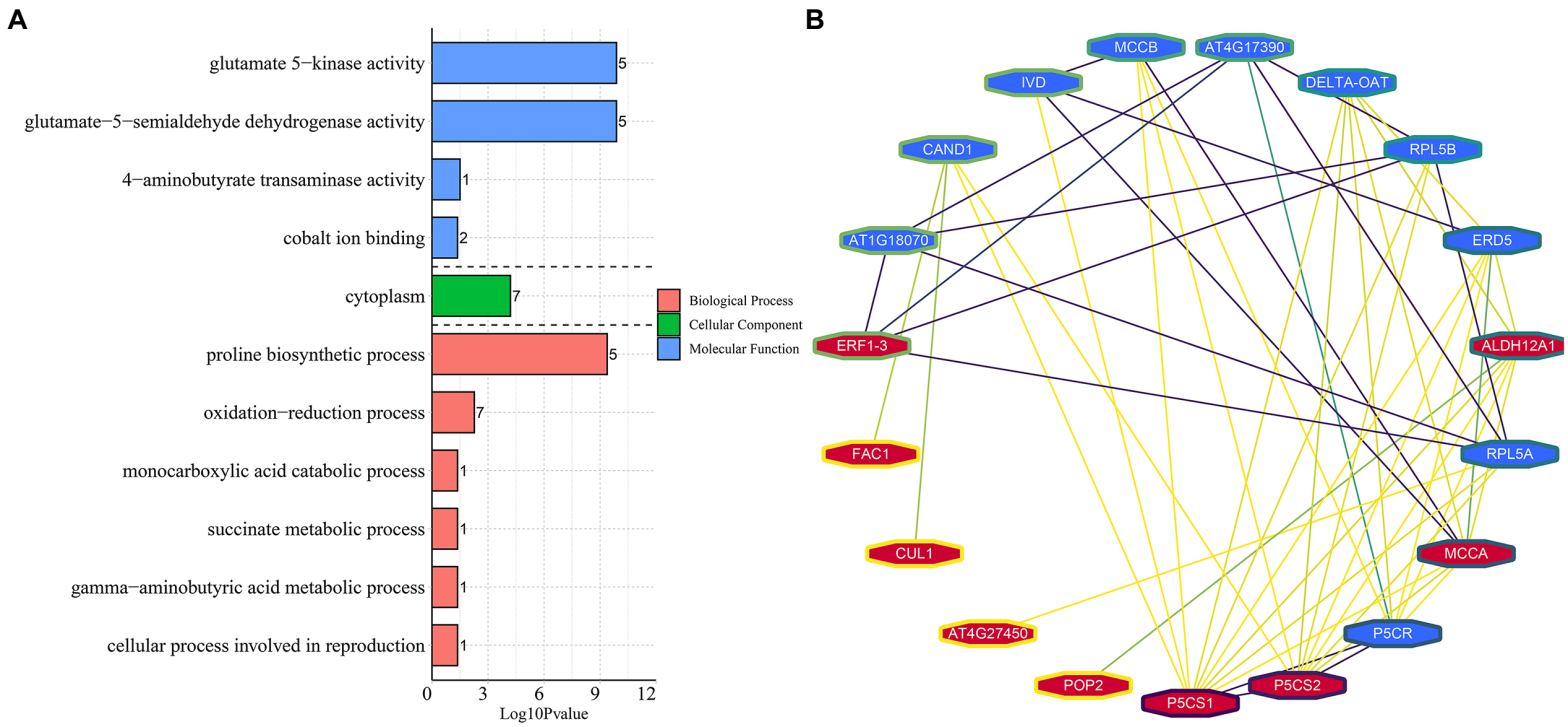
Functional enrichment **(A)** and protein–protein interaction analysis of 29 hub DEGs **(B)**.

To detect the conserved modules of DEGs under multiple abiotic stresses, the consensus expression profile modules of single drought, cold, and co-occurrence of cold-drought stress were performed by combining the results we published previously (Min et al., 2020b,c). All DEGs could be clustered into six significantly enriched trends (*p* < 0.05) in leaves and roots, including two upregulated patterns (profile 19 and 17), two downregulated patterns (profile 0 and 2), one profile only upregulated under cold and cold-drought stress (profile 12), and one profile only downregulated under cold and cold-drought stress (profile 7; Figures 7A,B). Compared with other profiles, we paid more attention to profile 19, because these DEGs were upregulated under all abiotic conditions. To further discover the key genes’ response to multiple stresses, 29 co-induced DEGs identified in both tissues were selected as hub genes (Figure 7C). The top two hub genes were *Delta-1-pyrroline-5-carboxylate synthase B* (*P5CSB*) and *Late embryogenesis abundant* (*LEA2*). Additionally, some proteins, including ABA-responsive protein ABR18, ERF1-3, monosaccharide-sensing protein 2, MtN21/EamA-like transporter protein, phosphatase 2C protein, and topless-related protein 2, and two hypothetical proteins were also played some crucial roles in the response to multiple abiotic stresses in whole plants. Functional enrichment results demonstrated that 29 co-induced DEGs were relevant to “glutamate-5-semialdehyde dehydrogenase activity,” “glutamate 5-kinase activity,” “proline biosynthetic process,” “cytoplasm,” and “oxidation–reduction process” (Figure 8A). Additionally, we also found that the arginine and proline metabolism pathway (ko00330), and biosynthesis of amino acids (ko01230) were the most enriched pathways among the hub genes (Supplementary Table S10).

To explore functional interactions among 29 hub DEGs obtained from common vetch, STRING analysis was conducted to present known protein coexpression by BLASTp homology searches against the *Arabidopsis* genome, and 19 unique orthologues were identified. Furthermore, Cytoscape was used to display the most overrepresented DEGs (Figure 8B). In the protein–protein interaction network, nearly half of them were enzyme proteins, including four hydrolases [*AtPP2C33* (*F01.PB22768*), *UBP2* (*F01.PB24895*), *MEE13.8* (*F01.PB29312*), *FAC1* (*F01.PB21275*)], three protein kinase genes [*AT5G58950* (*F01.PB17503*), *P5CS2* (*F01.PB22755*), *P5CS1* (*F01.PB17947*)], one oxidoreductase [*ALDH12A* (*F01.PB33234*)], one aminotransferase [*POP2* (*F01.PB20922*)] and, one ligase [*MCCA* (*F01.PB33120*)].

### Functional validation of hub genes

The enrichment result showed that “arginine and proline metabolism” and “plant hormone signal transduction” were the most co-enriched pathways in leaves, roots, and co-induced DEGs, respectively (Figure 5). Previous research has shown that *Delta l-pyrroline-5-carboxylate synthetase* (*P5CS5*) catalyzes is the first and second steps in proline biosynthesis. In our study, we find that five *VsP5CS2* amino acid kinases (*F01.PB22519*, *F01.PB13370*, *F01.PB22755*, *F01.PB17947*, and *F01.PB25217*) were upregulated under all abiotic conditions in both leaves and roots. Among them, the expression of *F01.PB22519* near zero under normal conditions, but significantly upregulated in both tissues under different abiotic stress. In addition, *F01.PB9597* (*VsUMAMIT*: Usually Multiple Acids Move in and out Transporters) was identified as an amino acid and auxin transporter, which was upregulated in all abiotic stresses, especially cold-drought combined stress. Furthermore, F01.PB3916 (VsPM19) is an ABA-induced plasma membrane protein, previous research has shown that an increased level of this protein leads to greater tolerance to low temperature (Koike et al., 1997). The rice homolog, OsPM19L1, is induced by osmotic stress and may be associated with stress tolerance through an ABA-dependent pathway (Chen et al., 2015). But whether *F01.PB9597* and *F01.PB3916* responds to multiple abiotic stresses remains unclear. Therefore, *F01.PB9597*, *F01.PB22519*, and *F01.PB3916* were selected as candidate genes for further studies. To verify three hub genes in response to individual and simultaneous stresses, we overexpressed them in the *S. cerevisiae* yeast strain INVSc1.

**Figure 9:**
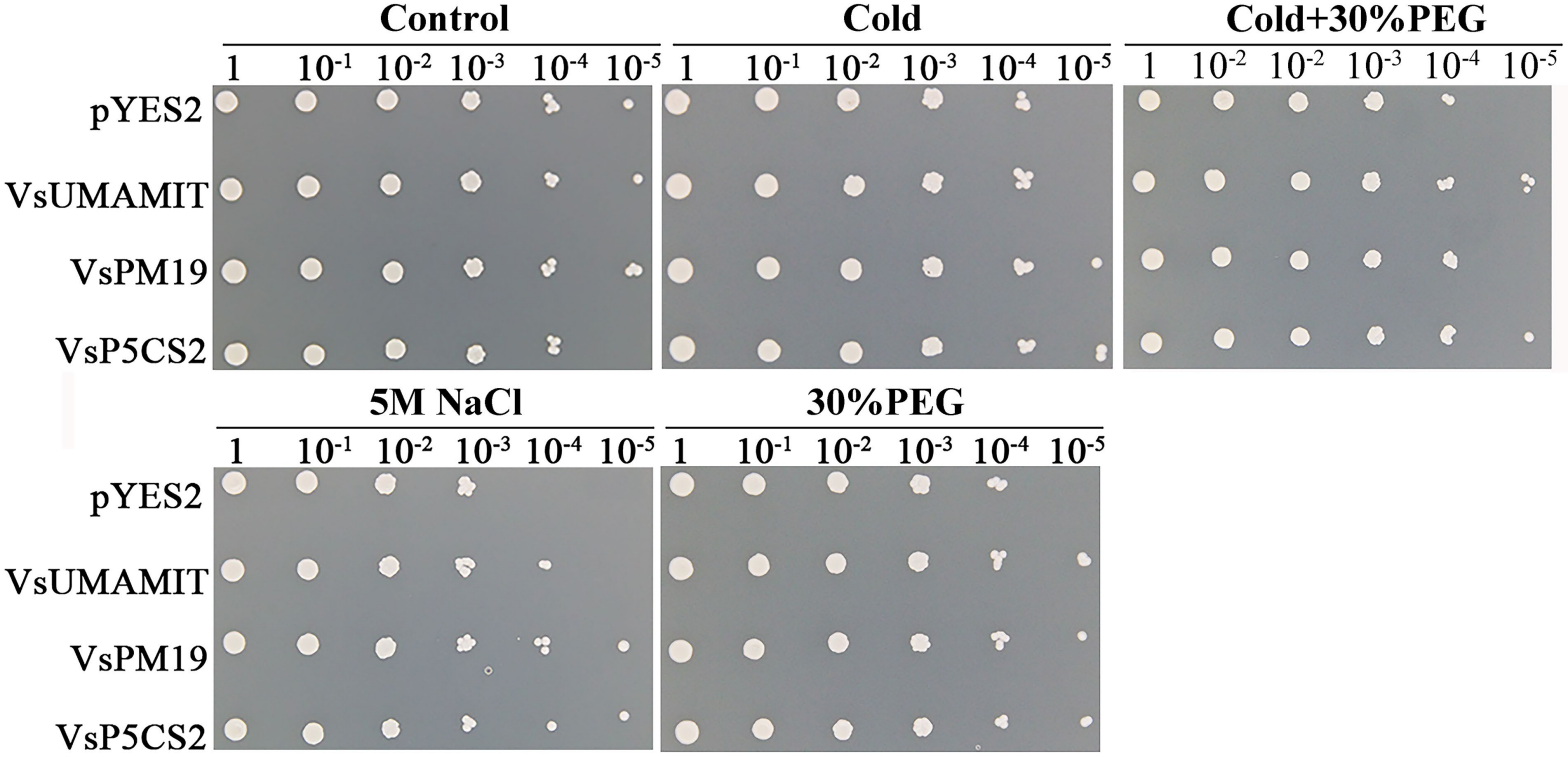
The expression of pYES2, pYES2-VsUMAMIT, pYES2-VsPM19, and pYES2-VsP5CS2 in INVSc1 cells induced under cold (−20°C), 5 M NaCl, 30% PEG, and cold + 30% PEG stresses.

Under normal conditions, the *pYES2-VsPM19* recombinant plasmid grows better than the empty yeast cells, while *pYES2-VsP5CS2* were not growing well as the control. When the transgenic yeast was treated with 5 M NaCl, 30% PEG, cold (−20°C), and cold-30%PEG combined stress for 36 h, the empty and recombinant plasmid cells showed different growth states. Under single abiotic stress, *pYES2-VsPM19* and *pYES2-VsP5CS2* can still grow in 10^−5^, but the *pYES2* plasmid transgenic yeast cells were hardly grown in a 10^−4^ dilution. Although *pYES2-VsPM19* grew well than control under single abiotic stress, did not show a better cold-30%PEG combined stress tolerance, indicating that *VsPM19* could not enhance compound stress tolerance (Figure 9). Among different abiotic stresses, *VsUMAMIT* were more sensitive to 5 M NaCl, 30% PEG and cold-30%PEG stresses. These combined data indicated that *VsUMAMIT* and *VsP5CS2* could enhance abiotic tolerance, *VsPM19* can only increase the tolerance to single stress, the precise regulatory mechanism still needs further studies. These three hub genes that have been validated will provide a foundation for improving abiotic tolerance in future common vetch genetic manipulation.

## Discussion

The effects of cold and drought on transcriptional regulation have been broadly studied separately. Generally, a cascade of biochemical and molecular events was induced and led to similar responses in most individual abiotic stresses. However, in natural environments, simultaneous stress frequently occurs and always causes a more severe effect on plants. There may exist some unique, complex regulatory pathways under combined abiotic stress (Pandey et al., 2017). Additionally, involving combined stresses, plant leaves, and roots can respond to these changes in various ways. Thus, at the molecular level, understanding common vetch responses to cold-drought combined stresses among leaves and roots will be necessary to identify key molecular targets and adaptive mechanisms.

### Common vetch responses to combined cold and drought stress are also unique

Our previous studies showed that the length and fresh weight of the aboveground and underground parts were significantly reduced from those of the control groups (*p* < 0.05), especially the fresh weight, which is 32% less than that of the control when the PEG concentration was up to 20% in cultivar “Lanjian No.1” (Min et al., 2020b). We also compared the physiological indicators of “Lanjian No.1” under 4°C treatment, and found that the fresh weight decreased by 4.4% after 24 h compared with that before treatment, and a significant increase in malondialdehyde and soluble sugar contents was detected at 24 h and maintained for at least 48 h under 4°C cold stress in leaves (Min et al., 2020c; Cui, 2021). At the molecular level, the responses of the leaves and roots of common vetch to combined cold and drought differ from those to each abiotic stress separately. Cold stress can alter the cell membrane fluidity and protein conformation, while drought induces hyperosmotic stress in plant cells (Zhang et al., 2022). However, cold and drought in combination alter photosynthesis, stomatal conductance, and respiration, distinctly from cold or drought alone. Under low temperature and drought conditions alone, more DEGs were identified in leaves, especially under low temperature, indicating that leaves are more sensitive to abiotic stress than roots. Consistent with previous studies, transcriptome sequencing showed that combined cold and drought stresses also have a greater impact on the leaves, and more upregulated DEGs were identified in maize and tomato leaves (Zhou et al., 2019; Guo et al., 2021).

Previous studies have indicated that plant responses to combined abiotic stress also exhibit some unique regulatory mechanisms and cannot be inferred from individual stresses. For example, a transcriptomic study in common vetch showed that Ca^2+^ signaling, hormonal signaling, photosynthesis signaling, and redox pathways play important roles through CBF-dependent or CBF-independent transcriptional mechanisms to enhance cold resistance (Min et al., 2020c). Under single drought stress alone, hormonal signaling, starch and sucrose metabolism, and arginine and proline metabolism were the most enriched pathways among leaves and roots (Min et al., 2020b). Thus, a comprehensive understanding is essential for improving common vetch resistance to combined stress. Under combined cold and drought stress, ABC transporters were the most enriched pathway among leaves and roots, followed by arginine and proline metabolism, circadian rhythm-plant, plant hormone signal transduction, starch, and sucrose metabolism (Figure 5). Cutin, suberin and wax biosynthesis, and glucosinolate biosynthesis were the most enriched pathways in leaves and roots, respectively. ABC transporters bind ATP and hydrolyze ATP, which are essential for plant growth, development, and adaptation to the harsh environment (Dahuja et al., 2021). In this study, 18 *ABCB*s and three *ABCC*s were identified as having different (positive or negative), or tissue-specific expression patterns. Some studies have shown that the expression of *ABC* genes may regulate wax and cutin transport in plant leaves, and further affect plant responses to stress, including cold and drought. In rice, compared with the wild type, the wax crystals disappeared in osabcg9-2 mutant leaves, and cuticular wax was diminished by 53%, increased leaf chlorophyll leaching, and became more sensitive to drought stress in *osabcg9-1* mutant plants (Nguyen et al., 2018). In *Arabidopsis*, *AtABCG11*, *AtABCG12*, *AtABCG13*, and *AtABCG32* are involved in cuticular wax and cutin monomer transportation (Yeats and Rose, 2013). Under natural conditions, common vetch always faces combined abiotic stresses, so it is important to understand how common and stress-specific response pathways interact.

In general, combined cold and drought stress is more likely to disrupt the PS-II function of aboveground tissues in plants, and then significantly reduce photosynthetic activity (Guo et al., 2021). Upon abiotic stress, abiotic-stabilized phyB could promote plant tolerance by controlling the expression of some stress-responsive genes, enhancing photosynthetic efficiency (Zhang et al., 2022). The *CAB*s are the main functional components of the light-harvesting complex (LHC) in higher plants. *CAB* genes are classified into 10 gene families, and six *CAB* families, including *LHCb1*, *LHCb2*, *LHCb3*, *LHCb4*, *LHCb5*, and *LHCb6*, are related to photosystem II (PS II; Plöchinger et al., 2016). CAB genes have been identified to play a crucial role in the response to light intensity, drought, low temperature, and salinity stress. Wang et al. (2017) performed a proteomic analysis of tea leaves and found that the expression of *CAB* genes increased dramatically during drought stress (Wang et al., 2017). *Arabidopsis LHCb4* mutant plants have a disrupted PS II macrostructure and are defective in photoprotection, proving that *LHCb4* plays a unique role among PS II antenna proteins and photoprotection (de Bianchi et al., 2011). Abundant chlorophyll a-b binding proteins significantly contributed to the “photosynthesis” pathway, and all of them were significantly upregulated, indicating that photosynthesis was enhanced under abiotic stress in common vetch leaves.

*Dehydrin* (*DHN*) genes are highly hydrophilic and are predominantly induced by cold, drought, and salt stresses (Shekhawat et al., 2011). Previous studies have suggested that *DHN* genes can promote the accumulation of osmotic material by protecting photosynthesis, enriching chlorophyll content, enhancing water retention capacity, and activating ROS detoxification (Singh et al., 2019). For instance, when rice seedlings are exposed to severe drought conditions resulting all *DHN* genes are upregulated in leaves and roots (Verma et al., 2017). In addition, the cold and drought tolerance was improved by overexpressing *DHN* genes in transgenic tobacco, cotton, and Sorghum plants, resulting in malondialdehyde, lipid peroxidation, relative electrolyte leakage, and water loss reduction (Bao et al., 2017; Guo et al., 2017; Halder et al., 2017; Kirungu et al., 2020). Our results emphasized that DHN proteins have a positive impact on common vetch cold-drought stress tolerance. In total, 20 differentially expressed *DHN* genes were enriched to the “Response to water” category, all *DHN* genes were upregulated in leaves, and seven of them were upregulated in both tissues. Furthermore, some of *VsDHNs*, including *F01.PB13924*, *F01.PB6182*, *F01.PB8865*, *F01.PB5084*, *F01.PB3012*, and *F01.PB47* were found to be negligible in leaves and/or roots under normal conditions and was significantly upregulated by more than 100-fold under combined treatments. Abundant *CAB* and *DHN* genes were significantly induced and enriched in common vetch leaves, confirming that *DHN*s may play a vital role in maintaining the photosynthetic rates under combined stress.

### TFs involved in the combined cold and drought stress

When plants face abiotic stress, many genes, especially transcription factors, can respond rapidly and regulate the expression of downstream functional genes. Previous studies found that C-repeat/dehydration-responsive element-binding (CBF/DREB) transcription factors could be activated by AP2/ERF, CAMTA, MYB, and nuclear factor Y (NF-Y), and play a key role in low temperatures and/or water deficit (Wang et al., 2012; Park et al., 2015; Hu et al., 2020). AP2/ERF family, as one of the largest gene families in plants, plays a diverse role in response to abiotic stresses and phytohormones and can control a wide network of downstream genes in the cellular signaling pathway (Faraji et al., 2020; Heidari et al., 2021). Consistent with previous studies, 18 and 22 AP2/ERF genes were identified under combined stress in common vetch leaves and roots. Among them, nine AP2/ERF genes were upregulated in both tissues. Some AP2/ERF genes showed tissue-specific expression patterns. Two *CAMTA* (*F01.PB25251*, *F01.PB28165*) were specifically upregulated in leaves, and one (*F01.PB23756*) was specifically downregulated in roots. Several transcription factor families have shown tissue-specific expression patterns were obtained. For example, *NF-YA* (*F01.PB13198*) was upregulated in leaves, and *NF-YB* (*F01.PB3279*) was downregulated in leaves. The CBF transcriptional level response is quite different between leaf and root tissues, which might interact and/or inhibit each other.

The NAC (NAM, ATAF, and CUC) TF family members play an important role in plant growth, development, and stress responses. In *Arabidopsis*, *ANAC019* (*AT1G52890.1*), *ANAC055* (*AT3G15500*), and *ANAC072* (*AT4G27410.2*) were induced by drought, salinity, and/or low temperature, and transgenic plants overexpressing either of them showed significantly increased stress tolerance compared to the wild type (Tran et al., 2004). In this study, the NAC transcription factors were observed to be the most significantly upregulated, including *ANAC072* orthologous *F01.PB6545*, *F01.PB8282*, and *F01.PB9565*, which are upregulated in both tissues. Dof (DNA binding with one finger) has been reported to participate in the regulation of gene expression in plant defense processes (Waqas et al., 2020). The expression levels of Dof transcription factors (*F01.PB31167* and *F01.PB10374*) changed significantly in both tissues in common vetch. Some of the previously uncharacterized abiotic transcription factors also identified in this study might represent novel regulators of combined stress tolerance in whole plants. Examples include HSI2-like 1 (*F01.PB35573*) and DBB (*F01.PB6342*) transcription factors.

## Conclusion

The present study designed to compare the transcription profiles of cold-drought combined stress between aboveground and underground tissues revealed the operation of mechanisms that (a) the responses of leaves and roots of common vetch to combined cold and drought differ from that subjected to single stress, as well as some unique genes related to stress tolerance, (b) cold-drought combined stress has a greater impact on leaves, and more upregulated DEGs were identified, (c) improve tolerance to combined stress by upregulating a large amount of chlorophyll a-b binding protein and water responsive genes (e.g., ECP40, DHN2 and Endochitinase A2) in leaves, many d response proteins (e.g., TMV resistance protein, NB-ARC domain disease resistance protein, ABA-responsive protein, and Endochitinase A2) in roots, and multifunctional enzymes (e.g., GABA-TP1, SUS2, and P5CS) were upregulated in both tissues. Additionally, the hub genes involved in the single and combined cold-drought stress were identified and functionally validated. Further metabonomic and transgenic studies are needed to study the effect of hub genes under both single and combined stresses.

## Data availability statement

The original contributions presented in the study are publicly available. This data can be found here: https://doi.org/10.6084/m9.figshare.20629467.v1.

## Author contributions

XM: methodology, software, validation, formal analysis, investigation, data curation, visualization, and writing—original draft. QW: formal analysis, supervision, and validation. ZW: resources and project administration. ZL: methodology, funding acquisition, and supervision. WL: investigation, methodology, conceptualization, funding acquisition, visualization, and project administration. All authors contributed to the article and approved the submitted version.

## Funding

This research was supported by the National Natural Science Foundation of China (32071862), the Natural Science Foundation of Gansu Province (20JR10RA622), and the Fundamental Research Funds for the Central Universities (lzujbky-2021-ct21, lzujbky-2021-it05, and lzujbky-2021-ct14), the Nature Science Foundation of the Jiangsu Higher Education Institution of China (22KJB230011), and the Natural Science Foundation of Jiangsu Province (BK20220583).

## Conflict of interest

The authors declare that the research was conducted in the absence of any commercial or financial relationships that could be construed as a potential conflict of interest.

## Publisher’s note

All claims expressed in this article are solely those of the authors and do not necessarily represent those of their affiliated organizations, or those of the publisher, the editors and the reviewers. Any product that may be evaluated in this article, or claim that may be made by its manufacturer, is not guaranteed or endorsed by the publisher.

## Footnotes

^1^ http://bowtie-bio.sourceforge.net/bowtie2/index.shtml

^2^ http://www.cs.cmu.edu/~jernst/stem

^3^ http://www.string-db.org/

^4^ https://cytoscape.org/

^5^ http://www.r-project.org

## Supplementary material

SUPPLEMENTARY FIGURE S1 Characteristics of novel transcripts. **(A)** The expression pattern distribution of novel transcripts. **(B)** The expression level of novel transcripts under cold-drought combined stress in leaves and roots.

SUPPLEMENTARY FIGURE S2 Validation of the expression profiles (log2-fold change) of 15 selected DEGs from leaves and roots using qRT–PCR. The green bars represent qRT-PCR-expressed leaves (left y-axis), and the blue trend lines represent the FPKM value changes of the DEGs (right y-axis).

SUPPLEMENTARY FIGURE S3 The expression (Log2-fold change) correlation of 15 DEGs based on RNA-Seq via qRT-PCR.

SUPPLEMENTARY FIGURE S4 The expression profile of 63 co-induced transcription factors in leaves and roots, respectively.
