# Supplementary figures and images for: Full-length transcriptional analysis reveals the complex relationship of leaves and roots in responses to cold-drought combined stress in common vetch
Source: Front Plant Sci. 2022 Sep 23;13:976094. doi: 10.3389/fpls.2022.976094 (PMC9538161; doi:10.3389/fpls.2022.976094)

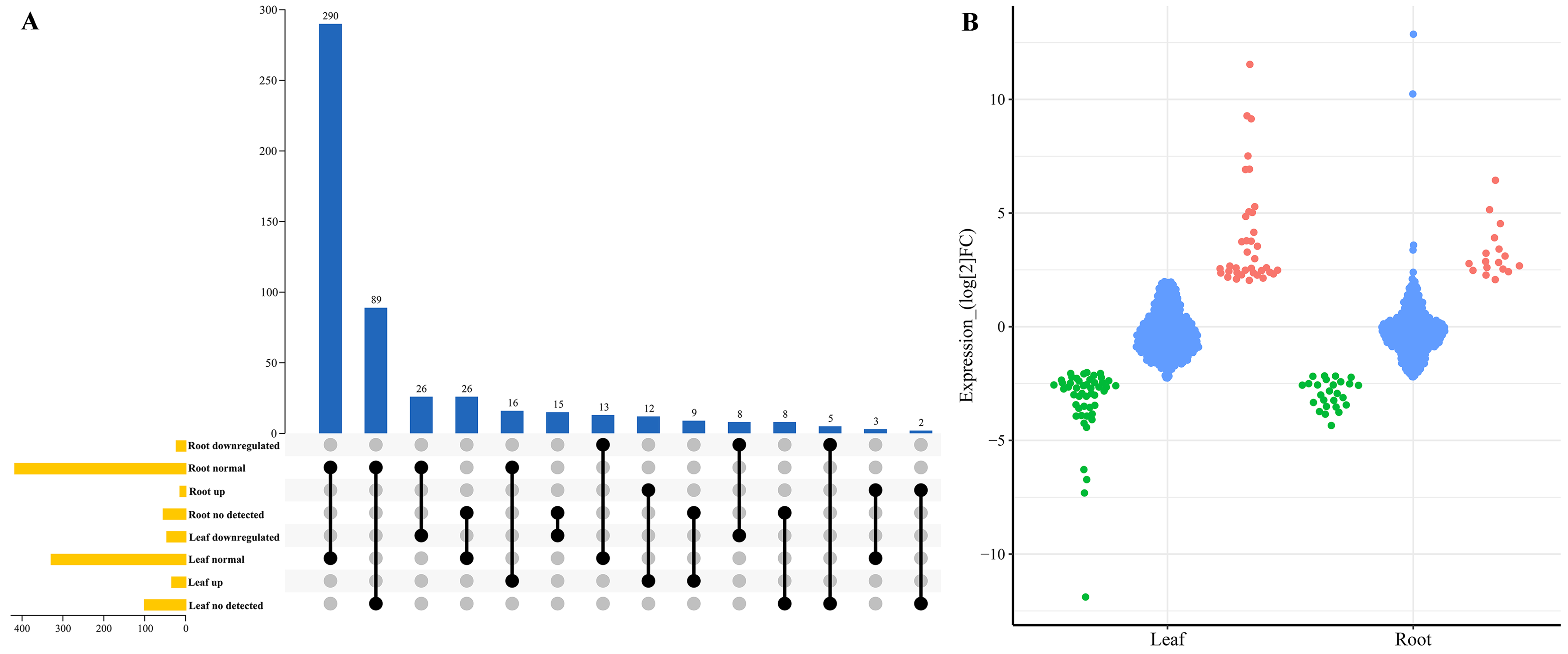

Supplement: SUPPLEMENTARY FIGURE S1 — Characteristics of novel transcripts. (A) The expression pattern distribution of novel transcripts. (B) The expression level of novel transcripts under cold-drought combined stress in leaves and roots. [file Image_1.TIF]

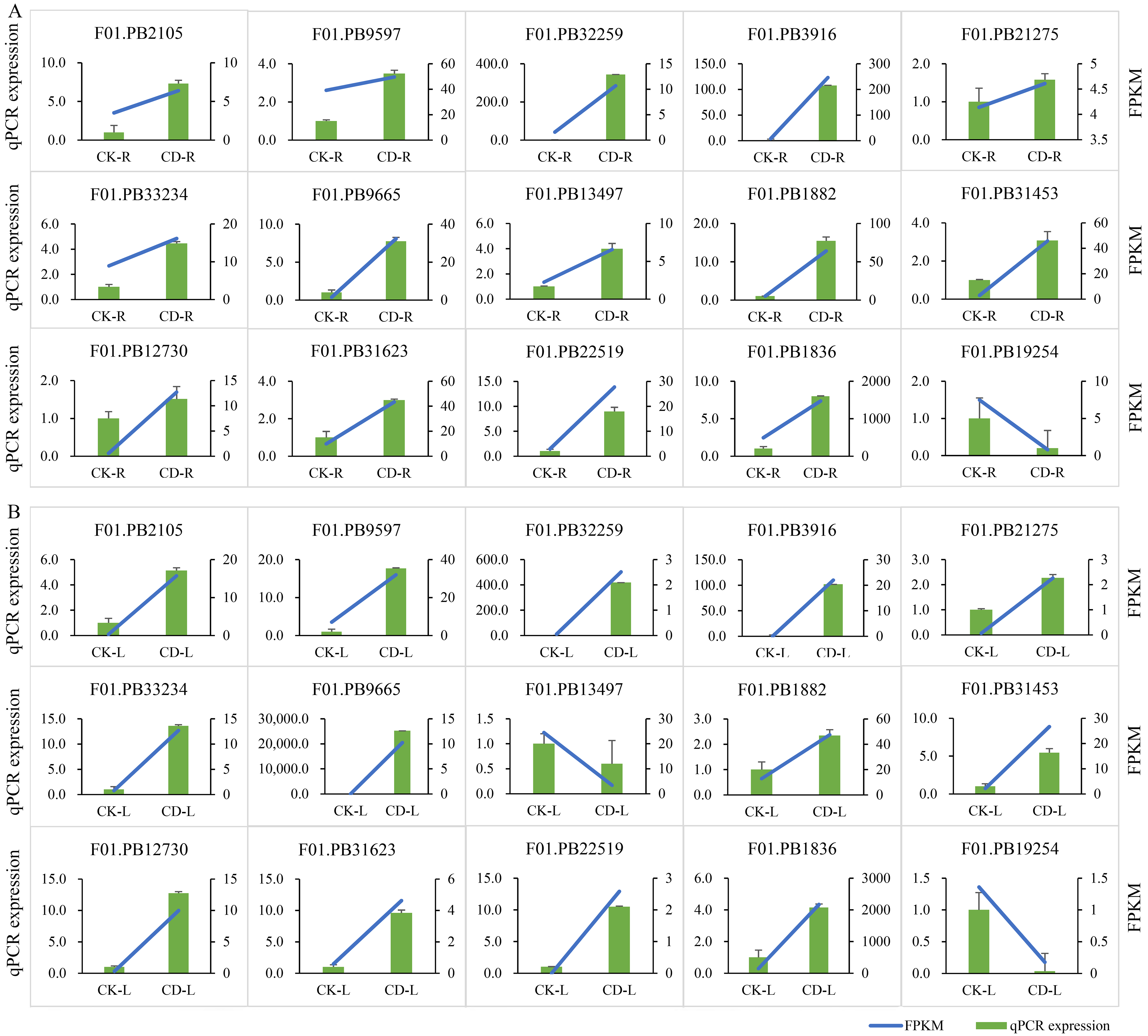

Supplement: SUPPLEMENTARY FIGURE S2 — Validation of the expression profiles (log2-fold change) of 15 selected DEGs from leaves and roots using qRT–PCR. The green bars represent qRT-PCR-expressed leaves (left y-axis), and the blue trend lines represent the FPKM value changes of the DEGs (right y-axis). [file Image_2.TIF]

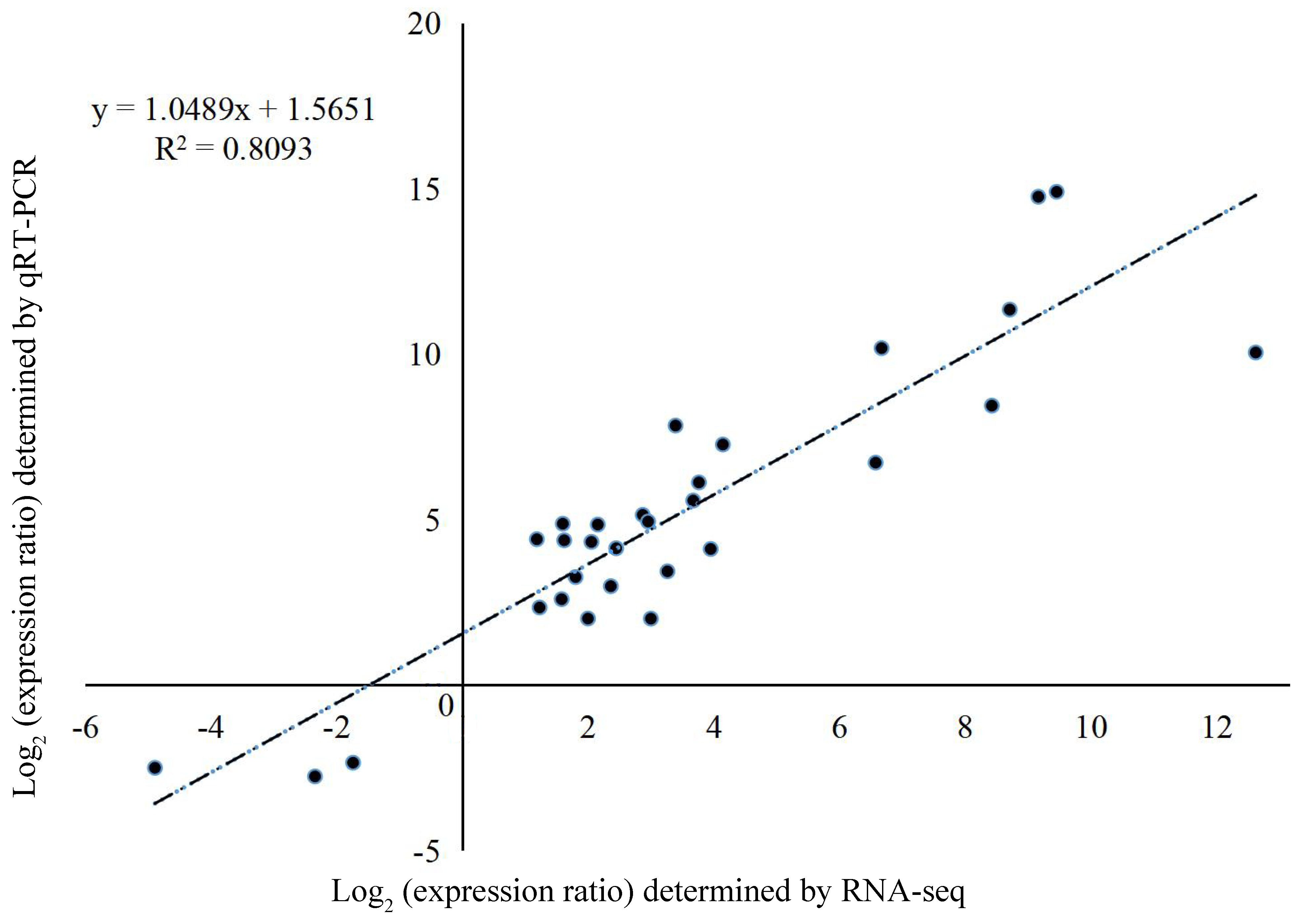

Supplement: SUPPLEMENTARY FIGURE S3 — The expression (Log2-fold change) correlation of 15 DEGs based on RNA-Seq via qRT-PCR. [file Image_3.TIF]

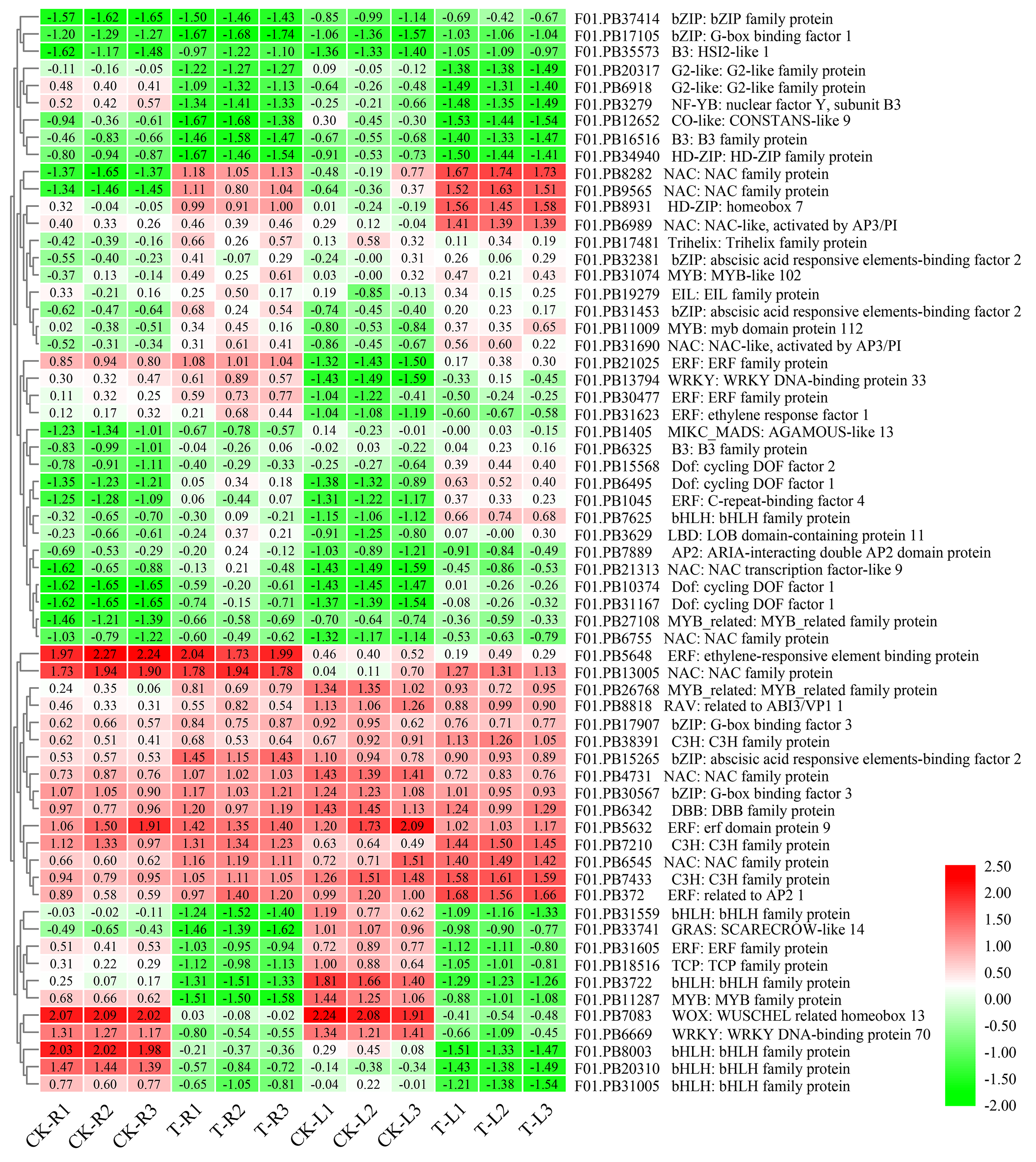

Supplement: SUPPLEMENTARY FIGURE S4 — The expression profile of 63 co-induced transcription factors in leaves and roots, respectively. [file Image_4.TIF]
