# Supplementary material for: Full-length transcriptional analysis reveals the complex relationship of leaves and roots in responses to cold-drought combined stress in common vetch
Source: Front Plant Sci. 2022 Sep 23;13:976094. doi: 10.3389/fpls.2022.976094 (PMC9538161; doi:10.3389/fpls.2022.976094)
Supplement: Supplementary file 5 [file Table_1.DOCX]

Table S1 Primers used for quantitative Real-time PCR and yeast assays in this study.

| Code | Gene name | Forward primer (5'->3') | Reverse primer (5'->3') |
| --- | --- | --- | --- |
| 1 | F01.PB2105 | ACACATGATTTTATTCGCAGAGGTT | ACTTTTCAACAACCTCATTGTCAG |
| 2 | F01.PB9597 | GGATGATTATGCAGGTGCCG | AGCAATTCCAATCCCCGCATA |
| 3 | F01.PB32259 | CCAACCAGATGATGGGCAAC | GGCTGTCTGATGCGTTTTGT |
| 4 | F01.PB3916 | TGGTGCAACCACGTTCTTCT | ACCCCATGGCTAGAGCAGTA |
| 5 | F01.PB21275 | ACCTTGCGCAGGTTGGATTA | CTCGCTGGAAAAACATGGGC |
| 6 | F01.PB33234 | GCCGCTAGGGTTTCTTCTCT | TCTACTGTGGCAGCAAGTGG |
| 7 | F01.PB9665 | GATGTTCCCGCACCTGACAT | AACCACAGCAGGAGAATGACC |
| 8 | F01.PB13497 | GCCCTCCGAGAGAAAAACGA | GCTTTCGAGCAATCTCGCAC |
| 9 | F01.PB1882 | GCTCTTCCCGGTTCTACCAA | CCATCCACTGCAACTTTGACG |
| 10 | F01.PB31453 | GCTGTTCAAATTGGTGCCGT | CCCTTCCTTCCTCGCATACC |
| 11 | F01.PB12730 | TGATGGACATGGGGGCTTTC | CTCCAACCTCGTCGTCAACT |
| 12 | F01.PB31623 | TGATCAACGACGCAAACACC | TTTCCCCCACGGTCTTCTTC |
| 13 | F01.PB22519 | GGCCTTTATAGCGGTCCTCC | ATACCCCCTCTGCCCAATCT |
| 14 | F01.PB1836 | AGGAGTAGCAACCTCCGTCA | TGGAACCCACGACACCTTTT |
| 15 | F01.PB19254 | AACAACCGCTTTAGCAGGGA | CCCCAAACCCACTCCACAAT |
| 16 | F01.PB9597 | **CCC**AAGCTTATGTTTGTGACCTCTCTTATT | **CGC**GGATCCCTAAGAGCAATTCCAATCC |
| 17 | F01.PB3916 | **CGC**GGATCCATGGCTTCTGTGGGGAGAAATG | **CCG**GAATTCTTAAACACGAGTCGCAGGAAC |
| 18 | F01.PB22519 | **CCG**GAATTCATGAAAACAATGGAAGTTTTGC | **CCG**CTCGAGTTAAGCTCTTGCCTTCTCAGG |
| 19 | *Unigene68614* | GCTAAAGCATTGAACAACAAAAGA | GCAAAGTTTGTCCCTTCACC |

Note: underlined indicates the restriction site, bold indicates the protected base.
