# Supplementary material for: Full-length transcriptional analysis reveals the complex relationship of leaves and roots in responses to cold-drought combined stress in common vetch
Source: Front Plant Sci. 2022 Sep 23;13:976094. doi: 10.3389/fpls.2022.976094 (PMC9538161; doi:10.3389/fpls.2022.976094)
Supplement: Supplementary file 6 [file Table_2.DOCX]

Table S2 Summary of RNA sequencing reads information.

| cDNA libraries | Total reads | GC(%) | Q30(%) | Mapped reads (%) | Uniq mapped reads (%) | Multi mapped reads(%) |
| --- | --- | --- | --- | --- | --- | --- |
| CKL1 | 25,651,280 | 42.65 | 92.32 | 79.42 | 26.51 | 73.49 |
| CKL2 | 27,015,618 | 42.69 | 92.62 | 79.72 | 26.62 | 73.38 |
| CKL3 | 28,011,278 | 42.77 | 91.78 | 79.45 | 26.01 | 73.99 |
| CKR1 | 26,924,164 | 42.93 | 90.68 | 76.02 | 30.23 | 69.77 |
| CKR2 | 26,568,062 | 42.69 | 90.77 | 76.75 | 30.79 | 69.21 |
| CKR3 | 25,307,189 | 42.79 | 90.35 | 76.09 | 30.89 | 69.11 |
| CDL1 | 30,940,995 | 43.69 | 92.23 | 73.84 | 26.92 | 73.08 |
| CDL2 | 28,610,940 | 44.34 | 93.19 | 71.77 | 26.29 | 73.71 |
| CDL3 | 29,372,927 | 42.5 | 92.03 | 74.16 | 29.24 | 70.76 |
| CDR1 | 21,419,630 | 43.34 | 91.77 | 79.07 | 23.86 | 76.14 |
| CDR2 | 20,572,722 | 43.5 | 91.29 | 78.31 | 23.32 | 76.68 |
| CDR3 | 21,232,747 | 43.61 | 91.97 | 77.26 | 23.17 | 76.83 |
| Mean | 25,968,963 | 43.13 | 91.75 | 76.82 | 26.99 | 73.01 |
