# Supplementary material for: Full-length transcriptional analysis reveals the complex relationship of leaves and roots in responses to cold-drought combined stress in common vetch
Source: Front Plant Sci. 2022 Sep 23;13:976094. doi: 10.3389/fpls.2022.976094 (PMC9538161; doi:10.3389/fpls.2022.976094)
Supplement: Supplementary file 7 [file Table_3.DOCX]

Table S3 Success rate statistics of transcript annotation

| Term | Number of transcripts | Percentage |
| --- | --- | --- |
| Annotated in COG | 10390 | 33.82% |
| Annotated in GO | 19850 | 64.60% |
| Annotated in KEGG | 13439 | 43.74% |
| Annotated in KOG | 17388 | 56.59% |
| Annotated in PFAM | 25556 | 83.17% |
| Annotated in Swiss Prot | 23741 | 77.27% |
| Annotated in eggNOG | 28820 | 93.80% |
| Annotated in NR | 30125 | 98.04% |
| Annotated in only one database | 30250 | 98.45% |
| Annotated in all databases | 4879 | 15.88% |
| No database annotated | 521 | 1.70% |
